# Microneedle-based skin patch for blood-free rapid diagnostic testing

**DOI:** 10.1038/s41378-020-00206-1

**Published:** 2020-11-02

**Authors:** Xue Jiang, Peter B. Lillehoj

**Affiliations:** 1 Department of Mechanical Engineering, Rice University, Houston, TX 77005 USA; 2 Department of Bioengineering, Rice University, Houston, TX 77030 USA

**Keywords:** Engineering, Nanoscience and technology

## Abstract

Rapid diagnostic tests are one of the most commonly used tests to detect and screen for infectious diseases in the developing world. While these tests are simple, inexpensive, and readily available, they rely on finger-prick blood sampling, which requires trained medical personnel, poses risks of infection, and can complicate cooperation in young children, asymptomatic individuals, and communities with blood taboos. Here, we report a novel microneedle-based skin patch for the rapid detection of protein biomarkers in dermal interstitial fluid. Sample collection is facilitated by a hydrophilic hollow microneedle array that autonomously extracts and transports interstitial fluid to an antibody-based lateral flow test strip via surface tension for colorimetric antigen detection. We employ a simple gold enhancement treatment to enhance the detection sensitivity of this colloidal gold-based lateral flow assay and elucidate the underlying mechanism of this enhancement mechanism through experimental investigation. For proof-of-concept, this device was used to detect *Plasmodium falciparum* histidine-rich protein 2, a biomarker for malaria infection, which could be detected at concentrations as low as 8 ng/mL. Each test can be completed in <20 min and requires no equipment. To the best of our knowledge, this work is the first demonstration of a microneedle-based lateral flow assay for rapid protein detection in dermal interstitial fluid. In addition to its simplicity, minimally invasive nature, and low cost, this diagnostic device can be readily adapted to detect other protein biomarkers in interstitial fluid, making it a promising tool for point-of-care testing.

## Diagnostics: Microneedle skin patch for blood-free, rapid diagnostic testing

A simple, minimally invasive, low-cost microneedle skin patch has been developed that allows rapid detection of protein biomarkers in the dermal interstitial fluid (fluid surrounding skin cells). Rapid diagnostic tests are commonly used to detect and screen for infectious diseases in developing countries, but they rely on finger-prick blood sampling, which carries the risk of infection. Xue Jiang and Peter B. Lillehoj at Rice University, Houston, Texas, United States succeeded in creating a device that quickly collects dermal interstitial fluid using a microneedle array, and autonomously transports it to a lateral flow test strip. The authors verified their system with a biomarker for malaria infection, which could be detected at low concentrations. The test requires no equipment. The authors believe that their device has considerable potential for quickly detecting other protein biomarkers in resource-limited settings.

## Introduction

Infectious diseases are one of the leading causes of morbidity and mortality in the developing world^1^. In 2017, there were ~3 million deaths due to tuberculosis, HIV/AIDS, and malaria, most of which occurred in low- and middle-income countries^2–4^. One of the main roadblocks to disease control and elimination is the lack of simple, rapid, and low-cost tools for diagnostic testing^5^. While laboratory-based diagnostic tests, such as microscopy and molecular assays (polymerase chain reaction and enzyme-linked immunosorbent assay), exist for many infectious diseases, they are laborious, time-consuming, expensive, and rely on specialized equipment, making them ineffective for routine use in resource-limited settings. Rapid diagnostic tests (RDTs), such as lateral flow immunochromatographic assays, are simple, inexpensive, and readily available, making them promising tools for disease detection and screening in developing countries. For these reasons, RDTs are the most commonly used tests for detecting malaria infection worldwide. However, RDTs and laboratory-based methods both rely on blood sampling, which requires trained medical personnel and poses risks of infection or accidental disease transmission^6^. Furthermore, the invasive nature of blood sampling can complicate cooperation, especially in young children, asymptomatic individuals who are disinclined to be tested, and communities with blood taboos^7^. Efforts towards a blood-free diagnostic test for malaria have resulted in a urine dipstick for the detection of *Plasmodium falciparum* histidine-rich protein 2 (*Pf*HRP2)^8,9^; however, the moderate sensitivity of this method limits its usefulness for routine diagnostic testing. While *Pf*HRP2 has also been detected in the saliva of patients with malaria using ELISA^7,10^, its concentration in saliva is ~20× lower than in blood, which is well below the detection limit of malaria RDTs.

In the past few decades, much attention has been directed at the use of microneedles for minimally invasive transdermal drug and vaccine delivery^11^. Compared to hypodermic needles, microneedles avoid the nerves and vascular structures located in the deeper layers of the dermis, thereby significantly minimizing their associated pain and risks of infection^12^. Recently, the utility of microneedles for minimally invasive transdermal biosensing has also been reported^13^. Solid microneedles have been used for transdermal extraction of metabolites^14,15^ and protein biomarkers, including Dengue virus nonstructural protein-1^16^ and *Pf*HRP2^17^, in mice. While these platforms are capable of selective extraction of analytes, they require manual processing (e.g., desorption, pipetting, centrifugation) for recovery, concentration, and analysis. Prior studies have also demonstrated the extraction of dermal interstitial fluid using hollow glass and metal microneedles for subsequent biomolecular detection using conventional analytical methods (mass spectrometry, electrochemistry)^16,17^. Microneedle-based electrochemical sensors for measuring metabolites, such as glucose and lactate, in the dermal interstitial fluid have been demonstrated^18–21^; however, these platforms rely on electronic components (e.g., detectors, power source), which increases their size, complexity, and costs. Despite the recent progress made in microneedle-based biosensors, their current reliance on laborious sample processing or electronic hardware makes them poorly suited for use in resource-limited settings.

Here, we report for the first time a microneedle-based biosensing platform for rapid, colorimetric detection of protein biomarkers. This novel device integrates a hollow microneedle array with a colloid gold-based lateral flow immunoassay on a disposable skin patch. We show the ability of this device to effectively penetrate the skin and autonomously extract and transport liquid samples via surface tension using in vitro skin models. We also demonstrate the detection of *Pf*HRP2 in the dermal interstitial fluid at concentrations as low as 8 ng/mL. Lastly, we demonstrate proof of principle by using this patch to detect *Pf*HRP2 in young porcine cadavers, which was completed in <20 min and required no equipment.

## Results

### Design of the skin patch

**Fig. 1:**
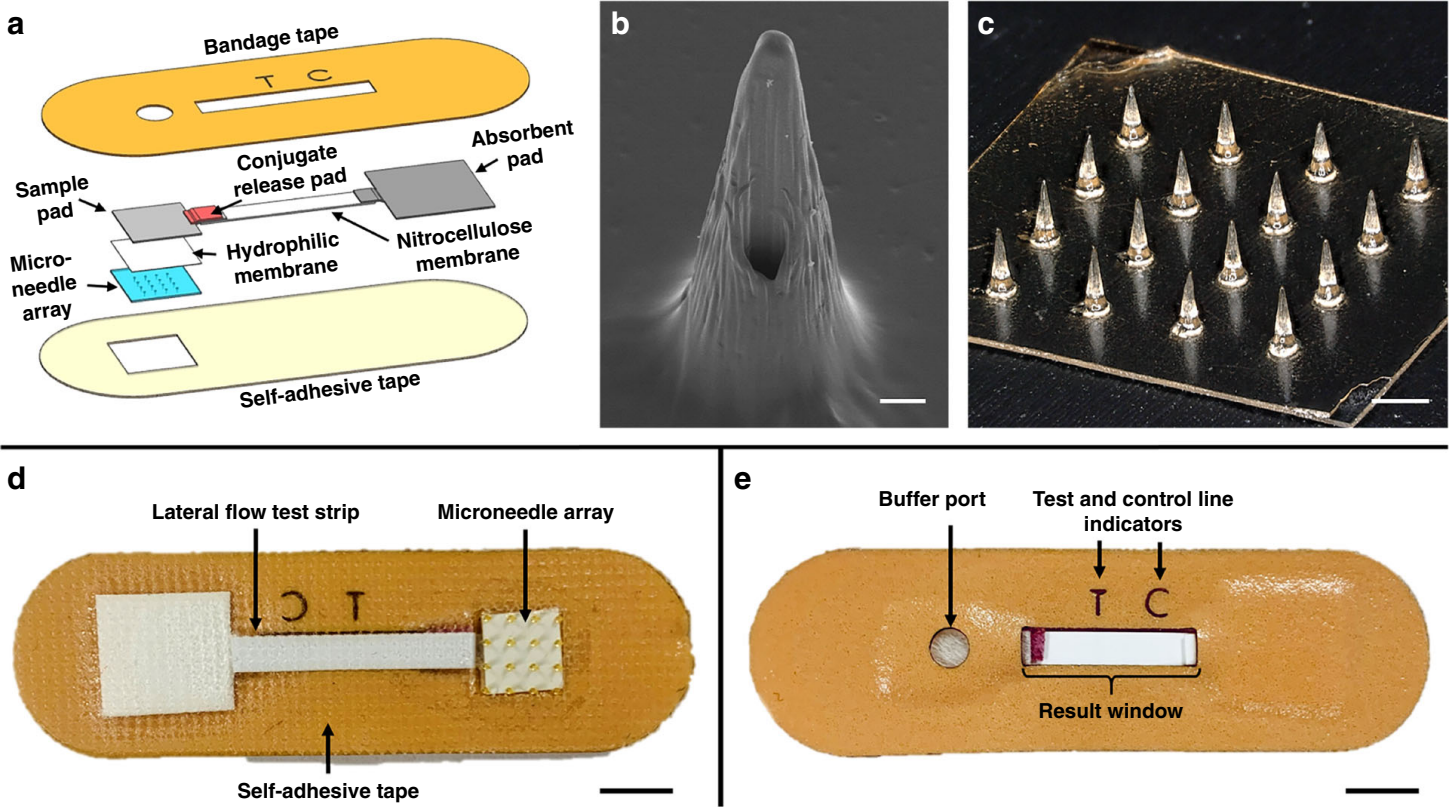
Design of the skin patch. **a** Exploded view of the patch depicting the microneedle array and components of the colloidal gold-based lateral flow immunoassay. **b** Scanning electron micrograph of a single microneedle. Scale bar, 100 µm. **c** Optical micrograph of the microneedle array at ×20 magnification. Scale bar, 1 mm. The underside (**d**) and topside (**e**) views of the assembled patch. Scale bars, 6 mm

The device consists of a hollow polymeric microneedle array integrated with a colloidal gold-based lateral flow immunoassay encased within a self-adhesive patch (Fig. 1a). The microneedle array is comprised of hollow microneedles made from polymerized SU-8 photoresist on a flexible polyethylene terephthalate (PET) substrate. Polymerized SU-8 exhibits excellent biocompatibility with no cytotoxicity effects and minimal interaction in tissue^22^, making it well suited for in vivo skin insertion. The microneedles were designed to safely penetrate human skin with minimal pain and extract dermal interstitial fluid for subsequent protein detection. Dermal interstitial fluid is located in the dermis, which is situated directly beneath the epidermis. The preferred application site for the patch is the upper anterior forearm due to its ease of access and lack of body hair^23^. The thickness of the skin in the forearm ranges from 800 to 1000 µm^24^. Studies evaluating the effect of microneedle design on pain in humans^12^ showed that microneedles ≤700 µm in length resulted in significantly less pain than that resulting from a 26-gauge hypodermic needle. Furthermore, sharp microneedles (tip angles ≤20°) generated the least amount of pain compared with microneedles with tip angles >20°, and there was no significant difference in pain for 700-µm-long needles having widths of 160, 245, or 465 µm. Based on these results, we designed the microneedle array to minimize pain during skin insertion while facilitating sample collection. Each microneedle is 750 ± 25 µm in length with a base diameter of 375 ± 25 µm and a hole diameter of 80 ± 10 µm. The hole is offset 80 µm from the central axis of the needle, resulting in a sharp lancet point geometry with a tip angle of ~14° (Fig. 1b). This tip geometry has been shown to minimize the force required for skin penetration, making it similar to that of a conventional 26-gauge hypodermic needle^25^. Microneedles are configured in a 4 × 4, two-dimensional array to increase the rate of sample extraction, with a needle-to-needle spacing of 2 mm (Fig. 1c). The overall size of the array is 7 × 7 mm, resulting in a miniature device footprint.

The microneedle array is affixed to a lateral flow test strip, which is based on a conventional lateral flow immunochromatographic assay consisting of a cellulose sample pad, a glass fiber conjugate release pad, cellulose absorbent pad, and nitrocellulose membrane on a polyvinyl chloride backing card. The conjugate release pad contains anti-*Pf*HRP2 IgG antibodies labeled with gold nanoparticles (AuNPs), and the nitrocellulose membrane contains immobilized anti-*Pf*HRP2 IgM antibodies and anti-mouse IgG H&L antibodies representing the test line and control line, respectively. Upon applying the patch to the skin, the microneedles penetrate the skin and extract dermal interstitial fluid via surface tension. The sample region is designed to allow for the extracted interstitial fluid to be autonomously transported from the microneedles to the backside of the sample pad via a hydrophilic membrane for subsequent passage through the lateral flow test strip. After an ~15-min sample collection period, a drop of saline solution (PBS) is dispensed in the buffer port. The buffer solution serves as a flushing agent and facilitates the transport of the interstitial fluid through the test strip after it has been collected in the sample pad. If *Pf*HRP2 is present in the sample, it will bind to AuNP–IgG conjugates and migrate toward the test line, where they will be captured to generate a red line (denoting a positive result). Uncaptured AuNP–IgG conjugates will subsequently bind to the control line to generate a second red line, verifying the test result. If the sample does not contain *Pf*HRP2, the AuNP–IgG conjugates will only bind to the control line and generate a single red line (denoting a negative result).

The microneedle array-lateral flow test strip assembly is sandwiched between two layers of medical-grade Nexcare^TM^ tape (the top layer is opaque, and the bottom layer is transparent), as shown in Fig. 1d. The underside of the patch contains a cutout for the microneedle array and allows for the entire assembly to be fully encased within the patch, eliminating potential hazards associated with reagent leakage. The topside of the patch contains cutouts for the buffer port, result window, and test (“T”) and control (“C”) line indicators (Fig. 1e). The self-adhesive backing of the patch allows for it to remain in place on the skin during testing.

### Skin penetration performance

We first characterized the mechanical strength of the microneedle array to determine its capacity to penetrate human skin. Force-displacement curves of the microneedle arrays subjected to mechanical compression are shown in Supplementary Fig. S1. The vast majority of microneedles in the arrays do not exhibit any plastic deformation after compression testing and remain in the elastic region during the entire test, which is verified by the nearly linear force vs. the displacement curve. The slight bend in the response curve (occurring at ~0.2 N/needle) is due to the deformation of a few microneedles that are slightly longer (tens of microns) than the rest of the microneedles in the array. It has been previously reported that the force required to penetrate human skin is 0.08 N/needle^26^; therefore, our microneedles will not exhibit any deformation during skin penetration. Furthermore, the yield strength of the microneedle array is at least 17 N ± 1 N, which denotes a minimum safety factor of 12.5, ensuring that the microneedles will not break during skin penetration, thereby eliminating potential risks associated with microneedle failure.

**Fig. 2:**
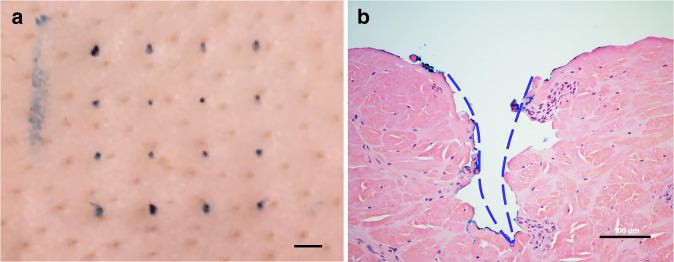
Skin penetration performance of the microneedle array. **a** Trypan blue staining of microneedle insertion wounds in cadaver porcine skin. Scale bar, 1 mm. **b** H&E-stained section of porcine skin penetrated by the microneedle array. The insertion wound is indicated by the blue dashed line. Scale bar, 100 μm

Next, we assessed the skin penetration capability of our microneedle array using porcine skin, which is anatomically and biochemically similar to human skin^27^. As shown in Fig. 2a, a confined insertion wound is generated by each microneedle with no impact on the surrounding skin. Histological analysis was also performed to evaluate the effects of microneedle penetration in skin tissue. As shown in the H&E-stained section of porcine skin (Fig. 2b), each insertion site is characterized by a conical penetration cavity that pierces through the epidermis. The depth of the cavity is ~420 μm, which enables the extraction of dermal interstitial fluid while avoiding the nerves and vascular structures located in the deep layers of the dermis. Optical micrographs of the microneedle array before and after skin insertion were also obtained to evaluate microneedle integrity. As shown in Supplementary Fig. S2, the microneedles exhibit no discernable deformation or damage following skin penetration. These collective results validate the effectiveness of the microneedle array in safely penetrating skin for interstitial fluid collection and suggest that it will cause no bleeding and minimal pain when inserted into the skin.

### Capillary-based sample collection

**Fig. 3:**
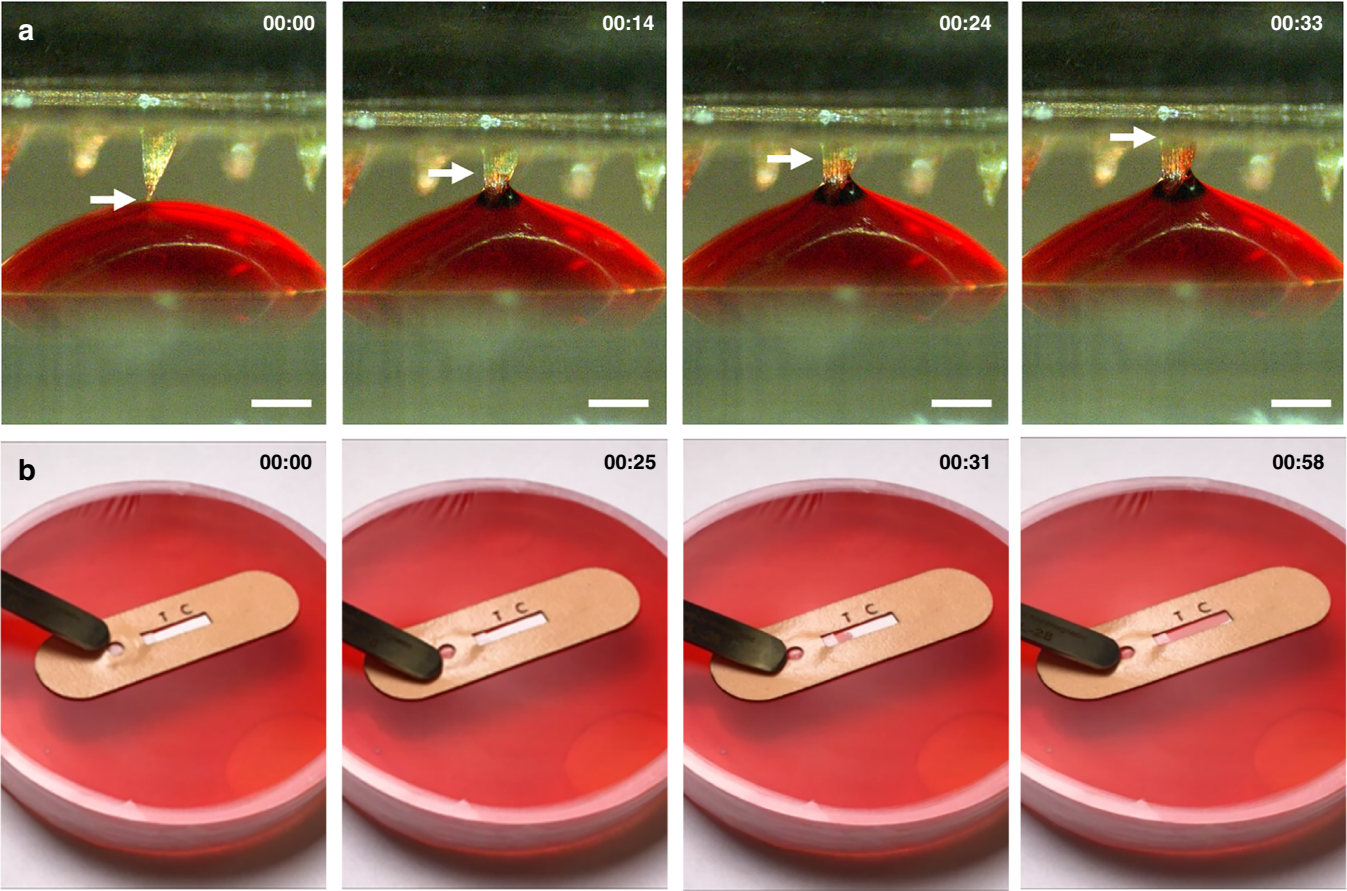
Fluid extraction capability of the microneedle array and skin patch. **a** Sequential still frame images showing a droplet of red dye being autonomously wicked by the microneedle array. The white arrow indicates the position of the liquid inside the needle. Scale bars, 500 μm. **b** Sequential still frame images showing capillary-based liquid extraction and transport through the skin patch in an artificial skin model. Timestamps (min:sec) are located in the upper right corners

While polymerized SU-8 offers excellent biocompatibility and exceptional mechanical properties advantageous for skin penetration, its mild hydrophobicity (water contact angle ~80 °C)^28^ hinders its capacity for capillary-driven liquid transport. To address this issue, the surfaces of the microneedles were made hydrophilic through a UV/ozone surface treatment to facilitate sample collection and transport. UV/ozone exposure for 20 min has been shown to dramatically improve the surface wettability of polymerized SU-8 (water contact angle ~22°)^29^. We first evaluated the capability of UV/ozone-treated microneedles to wick liquids by inserting the tips of the microneedles into a droplet of red dye solution. As shown in Fig. 3a, the red solution is readily drawn into the microneedle tips upon initial contact and conveyed through the shafts of the microneedles via surface tension. On average, it takes <30 s for the liquid to fill the entire microneedle. Next, we applied the skin patch to an artificial skin model containing red dye solution. As shown in Fig. 3b, the solution is quickly extracted by the microneedle array and subsequently transported through the lateral flow test strip via surface tension. The time required for liquid to be extracted and wicked through the entire test strip is ~60 s.

### *Pf*HRP2 detection sensitivity and specificity

**Fig. 4:**
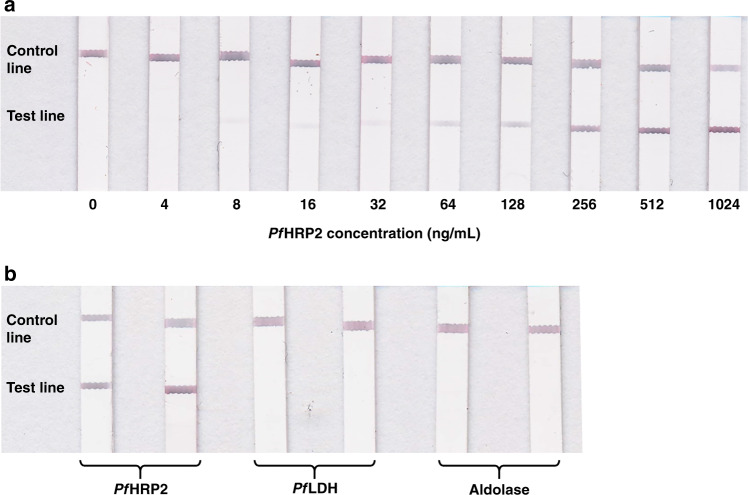
Sensitivity and specificity of the *Pf*HRP2 immunoassay. **a** Test results of interstitial fluid containing increasing concentrations of *Pf*HRP2. **b** Test results of interstitial fluid containing 1024 ng/mL *Pf*HRP2, *Pf*LDH, or Pan-*Plasmodium* aldolase

We assessed the sensitivity (i.e., the lower limit of detection) of the *Pf*HRP2 lateral flow immunoassay using dermal interstitial fluid simulant spiked with varying concentrations of recombinant *Pf*HRP2 antigen from 0 to 1024 ng/mL. We first evaluated the sensitivity of the assay without any sensitivity enhancement treatment. As shown in Supplementary Fig. S3a, the intensity of the test line is correlated with the *Pf*HRP2 concentration, which can be detected down to 16 ng/mL by the naked eye. We also investigated the use of a gold enhancement solution that can be applied directly to the test and control lines to enhance their contrast and improve the overall detection sensitivity. As shown in Fig. 4a, the intensity of the test lines is noticeably darker and remains correlated with the *Pf*HRP2 concentration, where the lowest detectable concentration that can be observed by the naked eye is 8 ng/mL. All of the measurements generated a dark control line, validating the test results. We also investigated the influence of the sample volume on the detection sensitivity by performing measurements using varying volumes (2, 5, 10, and 15 µL) of interstitial fluid simulant containing *Pf*HRP2 from 0 to 1024 ng/mL. As shown in Supplementary Fig. S4, the intensities of the test lines at all concentrations are similar for sample volumes ≥5 µL, where the lowest detectable concentration that can be observed by the naked eye is 8 ng/mL. The intensity of the test lines is noticeably lighter using a sample volume of 2 µL, resulting in a sensitivity of 8–32 ng/mL. These results suggest that high sensitivity measurements are achievable using sample volumes as low as 5 µL, which should be attainable in human skin using our skin patch.

We evaluated the specificity of the assay by performing measurements of interstitial fluid spiked with *Pf*HRP2, Pan-*Plasmodium* aldolase, or *Plasmodium falciparum* lactate dehydrogenase (*Pf*LDH), which are other common biomarkers for *Plasmodium falciparum* infection. As shown in Fig. 4b, only the *Pf*HRP2-containing sample generated “positive” test results, whereas the samples containing the irrelevant proteins generated “negative” results. These results show that the detection sensitivity of our device is up to 3× higher than that of malaria RDTs currently on the market (7–28 ng/mL)^30^ while exhibiting negligible cross-reactivity with other common *Plasmodium* protein biomarkers.

### Detection sensitivity enhancement

**Fig. 5:**
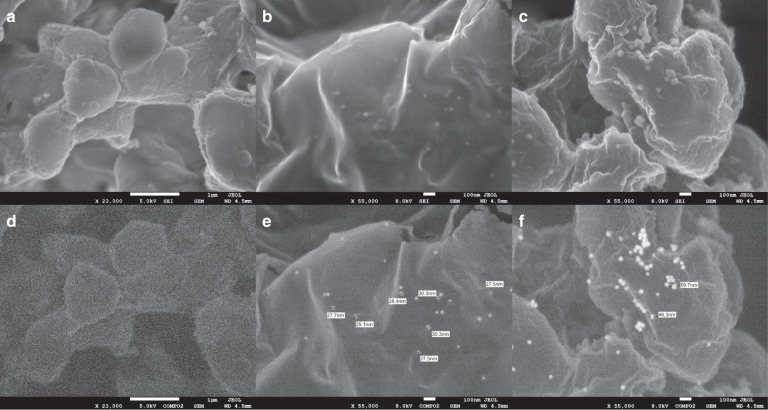
Characterization of AuNP–IgG conjugates with and without gold enhancement. Scanning electron micrographs of nitrocellulose paper obtained by SE imaging (**a**) and BSE imaging (**d**). Scale bars, 1 µm. Scanning electron micrographs of nitrocellulose paper coated with AuNP–IgG conjugates obtained by SE imaging (**b**) and BSE imaging (**e**). Scale bars, 100 nm. Scanning electron micrographs of nitrocellulose paper coated with AuNP–IgG conjugates with gold enhancement obtained by SE imaging (**c**) and BSE imaging (**f**). Scale bars, 100 nm

Several studies were performed to elucidate the mechanism by which gold enhancement treatment of AuNP–IgG conjugates results in improved detection sensitivity of our colloid gold-based immunoassay. We first characterized the morphology of AuNP–IgG conjugates immobilized on the test line of lateral flow test strips treated with or without gold enhancement solution using secondary electron (SE) and backscattered electron (BSE) imaging. SE and BSE images of the test strip without AuNP–IgG conjugates were also obtained and used as a blank control (Fig. 5a, d, respectively). As shown in Fig. 5b, e, untreated AuNPs are ~30 nm in diameter (consistent with the manufacturer’s specifications), whereas AuNPs treated with gold enhancement solution are enlarged by 50–100%, having diameters ranging from 45 to 60 nm (Fig. 5c, f). Absorbance spectra of AuNP–IgG conjugate solution treated with and without gold enhancement solution were also obtained and show a shift in the absorbance peak from 530 nm (without gold enhancement) to 562 nm (Supplementary Fig. S5a). The shift in the absorbance peak to a higher wavelength is consistent with the change in color of the test and control lines from red to purple (Supplementary Fig. S3)^31^. Finally, optical transmittance spectra were obtained from nitrocellulose membrane samples containing AuNP–IgG conjugates treated with or without gold enhancement solution. As shown in Supplementary Fig. S5b, samples treated with gold enhancement solution exhibit ~6% less light transmittance than that of untreated samples, thereby resulting in ~6% more light reflectance. Therefore, with the same concentration of AuNP–IgG conjugates immobilized on the test line, conjugates with large-sized AuNPs can generate more light scattering, effectively enhancing (i.e., darkening) the contrast of the test line^32^ and enabling lower concentrations of antigen to be detected.

### Proof-of-concept demonstration

**Fig. 6:**
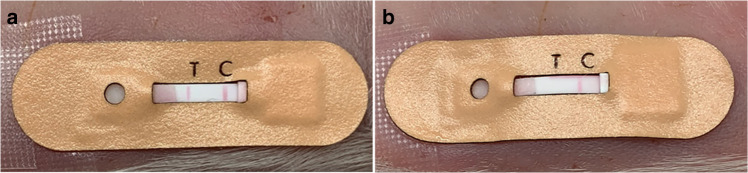
Proof-of-concept demonstration of the skin patch. Test results obtained from young pig cadavers dermally injected with (**a**) 1024 ng/mL *Pf*HRP2 or (**b**) PBS

To evaluate the functionality of the skin patch in vivo, we tested it on young pig cadavers. Cadavers for positive control measurements were dermally injected with 1024 ng/mL *Pf*HRP2, while cadavers for negative control measurements were injected with PBS. The patch was applied to a shaven section of skin preinjected with *Pf*HRP2 or PBS. On the cadaver injected with *Pf*HRP2, two red lines can clearly be observed in the readout window of the patch, indicating a “positive” result (Fig. 6a). In contrast, only the control line is observable in the result window of the patch applied to the cadaver injected with PBS, indicating a “negative” result (Fig. 6b). This proof-of-concept experiment validates the functionality of our skin patch to detect *Pf*HRP2 in vivo.

## Discussion

For most infectious diseases, laboratory-based diagnostic tests exist but suffer from various limitations that hinder their ability to be broadly employed in developing countries. For example, current methods for malaria diagnosis (thick and thin blood smears, ELISA, PCR, and RDTs) rely on trained medical personnel and blood sampling, making them poorly suited for mass screening, particularly in communities with blood taboos and large populations of asymptomatic infected individuals. To address these challenges, this skin patch employs a hollow microneedle array for minimally invasive, bloodless sampling of dermal interstitial fluid for subsequent protein detection. Proteomic analysis of dermal interstitial fluid has demonstrated nearly identical protein diversity compared with blood serum and plasma^33,34^, making it a promising source of disease biomarkers. It has been previously reported that *Pf*HRP2 can be detected in the dermal interstitial fluid of live mice tail vein injected with *Pf*HRP2^35^, suggesting that *Pf*HRP2 is also present in the dermal interstitial fluid of individuals with *Plasmodium falciparum* infection. Mean values for *Pf*HRP2 levels in the plasma of individuals with malaria infection range from 20 to 1750 ng/mL^36^. Based on other studies that showed levels of clinically relevant proteins (interleukins, C-reactive protein, histidine-rich glycoprotein, etc.) in dermal interstitial fluid and blood being comparable^37^, we also expect *Pf*HRP2 levels in dermal interstitial fluid and blood to be similar. Therefore, the sensitivity of this patch (8 ng/mL) makes it suitable for detecting varying serveries of malaria disease, including asymptomatic infection, uncomplicated malaria, and severe malaria. While this study focuses on the detection of *Pf*HRP2 due to its importance for diagnosing malaria infection, we envision that this patch can be used for the detection of other clinically relevant protein biomarkers that have already been identified and characterized in dermal interstitial fluid^37^, thereby further expanding the utility of this skin patch for point-of-care disease detection and screening.

Much of the existing work on minimally invasive transdermal biosensing employs solid microneedles due to the difficulty in fabricating hollow microneedle arrays. However, the use of solid microneedles for protein capture involves laborious sample processing to recover, concentrate, and analyze biomarkers, which complicates the overall testing process. Therefore, we have developed a unique fabrication process to generate hollow microneedle arrays from polymerized SU-8, which subsequently undergo UV/ozone treatment to make the surfaces hydrophilic. Based on the hydrophilic nature of the microneedles, dermal interstitial fluid is autonomously extracted upon skin penetration and transported through the lateral flow test strip via surface tension, eliminating the need for human involvement for sample collection. By doing so, the testing procedure using this skin patch is simpler than conventional RDTs, making it less dependent on trained medical personnel and enabling more widespread usage, particularly in the developing world. Prior studies have demonstrated the collection of interstitial fluid from human skin (1.5–4 µL/needle) using an array of hollow, metal microneedles with similar dimensions as our microneedles^17,33^. In another study, 1.1 µL (mean value, *n* = 15) of interstitial fluid was collected from human skin in ~5 min using a single hollow microneedle^38^. Based on these results, we hypothesize that each microneedle in our array is capable of collecting ~1 µL of interstitial fluid in human skin, resulting in a total sample volume of ~16 µL for the entire array, which is sufficient for sensitive biomarker detection using this device. By combining the simplicity, speed, and low cost of lateral flow technology with the bloodless nature of microneedle liquid sampling, this device has the potential to be a viable diagnostic tool for disease detection and screening in remote and resource-limited settings.

## Materials and methods

### Fabrication of hollow microneedle arrays

A schematic of the fabrication process is shown in Supplementary Fig. S6. A 100-µm-thick layer of polydimethylsiloxane (PDMS) (Dow Corning, MI) was spin-coated onto a 100-µm-thick PET film (McMaster-Carr, IL) and soft baked at 80 °C for 1.5 h. Two separate PDMS-PET assemblies were prepared. On one assembly, 50-µm holes configured in a 4 × 4 array were generated using a CO_2_ laser cutter (Universal Laser System, Inc., AZ). The other assembly was attached to the PET side of the laser-cut assembly, forming a four-layer PDMS-PET-PDMS-PET structure. One hundred nanometers of gold was thermally evaporated on the topside of the four-layer assembly, followed by the subsequent removal of the top PDMS layer. A total of 1000 µm of SU-8 2025 (MicroChem, MA) was weight-cast onto the three-layer assembly, baked at 95 °C for 12 h, and subjected to backside UV exposure (365 nm) through a custom photomask designed using AutoCAD software (Autodesk, CA). A 900-μm gap between the PET-PDMS-PET assembly and photomask resulted in the generation of tapered microneedles. The polymerized SU-8 substrate was baked at 55 °C for 1.5 h and placed in a 1-methoxy-2-propanol acetate solution (MicroChem, MA) for 3 h with gentle agitation, followed by ultrasonication for 20 min. Microneedle arrays were rinsed in isopropanol and deionized water, dried under a stream of purified N_2_, and left at room temperature overnight to fully dry. Arrays were exposed to a UV/ozone treatment (Novascan Technologies, IA) for 20 min to make the surfaces hydrophilic. The repeatability of this fabrication process is very good, where >80% of the arrays consist of microneedles with no defects. Of the remaining arrays with some minor defects, one or two microneedles may be slightly longer/shorter or exhibit slightly bent tips due to human errors during SU-8 coating or postexposure baking.

### Preparation of AuNP-conjugated IgG antibody

One milliliter of a 30-nm colloid gold solution (Sigma Aldrich, MO) was prepared at a concentration of 1.8 × 10^11^ particles/mL and centrifuged at 7000 × *g* for 10 min, followed by the removal of 660 µL of the supernatant. The precipitate was resuspended in the remaining supernatant via vortex mixing and ultrasonication. A solution of 0.82 μL of mouse anti-*Plasmodium falciparum* HRP2 IgG (ICL, Inc., OR) at a concentration of 9.28 mg/mL was added to the AuNP solution, vortexed for 30 s, gently agitated for 25 min, and incubated at room temperature for 20 min. The mixture was transferred to a new tube containing 7.5 mg of BSA powder, sonicated for 15 s, gently agitated for 25 min, and incubated at room temperature for 20 min. The mixture was centrifuged at 5000 × *g* for 20 min, the supernatant was removed, and the precipitate was resuspended in 200 μL of StabilBlock^®^ immunoassay stabilizer (Surmodics, Inc., MN) with 0.25% Tween-20. The AuNP–IgG conjugate solution was stored at 4 °C overnight prior to use.

### Preparation of the conjugate release pad

Untreated glass fiber strips (EMD Millipore, MA) were soaked in a solution containing 10% sucrose (Sigma Aldrich, MO), 2% bovine serum albumin (BSA) (Sigma Aldrich, MO), and 0.25% Tween-20 (Sigma Aldrich, MO) mixture for 1 h, and dried at 37 °C for 2 h. Strips were cut into 5 × 3 mm pads using a guillotine cutter (BioDot, CA), treated with 5 μL of AuNP–IgG conjugate solution, and dried at 37 °C for 2 h.

### Preparation of the nitrocellulose membrane

A 25-mm-wide nitrocellulose membrane (GE Healthcare, IL) was cut into 300-mm-long strips using a guillotine cutter. A solution of 200 μg/mL rabbit anti-mouse IgG H&L (Abcam, UK) and 800 µg/mL mouse anti-*Plasmodium falciparum* HRP2 IgM (ICL, Inc., OR) were dispensed on nitrocellulose membrane strips as control and test lines, respectively, using an automated liquid dispensing platform (BioDot, CA). The nitrocellulose membrane card was incubated at 37 °C for 2 h and cut into 3-mm-wide strips.

### Assembly of the lateral flow test strip

A schematic of the lateral flow test strip is shown in Fig. 1a. A 10 × 10 mm cellulose absorbent pad (EMD Millipore, MA) was attached to one side of the card with an ~3 mm overlap with the nitrocellulose membrane. The conjugate release pad and an 8 × 8 mm glass fiber sample pad (EMD Millipore, MA) were sequentially attached to the other side of the card with ~3 mm overlaps with the nitrocellulose membrane and conjugate pad, respectively. The absorbent and conjugate pads were cut from stock sheets using a CO_2_ laser cutter. Sample, absorbent, and conjugate pads were affixed to the nitrocellulose membrane using medical-grade, double-sided adhesive tape (3 M, MN). An 8 × 8 mm opening was generated in the adhesive tape under the sample pad using a CO_2_ laser cutter to provide access for the microneedle array.

### Assembly of the skin patch

The skin patch was assembled by first attaching the microneedle array to an 8 × 8 mm piece of Supor^®^-5000 hydrophilic membrane (Pall Corporation, NY), which was then attached to the backside of the sample pad of the lateral flow test strip. The microneedle array-lateral flow test strip assembly was affixed to a Band-Aid-sized piece of Nexcare^TM^ tape (3 M, MN) containing cutouts for the buffer port, result window, and test (“T”) and control (“C”) lines, which were generated using a CO_2_ laser cutter. A piece of transparent Nexcare^TM^ tape, containing a cutout for the microneedle array, was affixed to the underside of the device to fully enclose the lateral flow test strip and secure it within the patch.

### Mechanical testing of the microneedle array

The compression strength of the microneedle arrays was measured using a dynamic mechanical analysis machine (TA Instrument, DE). For each measurement, a single 4 × 4 microneedle array was affixed to the bottom plate using double-sided tape and compressed by the upper plate from 0 to 17 N at a rate of 0.5 N/min. Force-displacement curves obtained from four different microneedle arrays were combined and plotted as the mean data ± standard deviation using MATLAB. Force-displacement curves were normalized to set the initial position of the plate at zero displacement.

### Skin-insertion testing

Cadaver porcine skin was obtained from a local swine farm, rinsed in deionized water, shaved, and trimmed of fat using a scalpel (Cancer Diagnostics, Inc., NC). A 5 × 5 cm section was cut and secured to a wax block using pins. Microneedle arrays coated in a 0.4% Trypan Blue solution (Sigma Aldrich, MO) were pressed into the skin and removed after 5 min. Histological analysis was performed by soaking the skin sample and paraffin wax block in a 10% formalin solution (Cancer Diagnostics, Inc., NC) for 48 h, followed by fixing in paraffin, drying, cutting into 2-micron slices, and staining with hematoxylin and eosin (H&E) (Sigma Aldrich, MO). The morphology of the penetration holes was imaged and captured using a Nikon Eclipse LV100ND microscope and Nikon Digital Sight DS-Fi2 camera.

### Liquid-extraction testing

Liquid extraction testing of UV/ozone-treated microneedles was carried out by lowering the microneedle array into a droplet of red dye solution. The microneedle array was affixed to the motorized stage of a vertically positioned syringe pump (BD Scientific, NJ), and the stage was slowly lowered until the microneedle tips made contact with the droplet. Video recordings of liquid extraction were captured using a Fujifilm X-T20 digital camera with a Nikon 105-mm AF Micro- NIKKOR lens. We assessed the capacity of the skin patch to autonomously extract interstitial fluid using an artificial skin model. Briefly, 2% agar gel (Sigma Aldrich, MO) solution was boiled, poured into a 100-mm Petri dish, and cured at 4 °C overnight. The red dye solution was dispensed on top of the agar gel and covered by Parafilm (Bemis Company, Inc., WI), which was carefully stretched over the Petri dish to ensure removal of air bubbles. To initiate the test, the patch was placed on the artificial skin model and gently pressed using the blunt end of a pair of tweezers. Video recordings were captured using an iPhone XS. Postprocessing (i.e., frame extraction) of video recordings were performed using Microsoft Windows Live Movie Maker software.

### Evaluating *Pf*HRP2 immunoassay sensitivity and specificity

Recombinant *Pf*HRP2 antigen (CTK Biotech, CA) was serially diluted in dermal interstitial fluid simulant, which was prepared by diluting 10.3 mg/mL human serum albumin (Abcam, UK) in 10 mL of Tyrode’s solution (Sigma Aldrich, MO), as previously reported^39,40^ with minor modifications. For each measurement, 15 μL of the sample was dispensed on the sample pad, followed by 30 μL of PBS, which served as a flushing agent. After 5 min, 5 µL of GoldEnhance^TM^ Blots solution (Nanoprobes, Inc., NY) was applied to the test and control lines. For assay specificity testing, measurements were performed using dermal interstitial fluid simulant spiked with 1024 ng/mL *Pf*HRP2, Pan-*Plasmodium* aldolase (CTK Biotech, CA) or *Pf*LDH (CTK Biotech, CA). Images of the test results were obtained after 15 min using a Canon CanoScan 9000F scanner.

### Scanning electron microscopy of AuNP–IgG conjugates

Scanning electron micrographs of the test line of lateral flow immunoassays were obtained using a JEOL 7500F scanning electron microscope to characterize the morphology of AuNP–IgG conjugates with and without gold enhancement treatment. Lateral flow test strips were tested using 1024 ng/mL *Pf*HRP2, treated with 5 µL of GoldEnhance^TM^ Blots solution, rinsed in deionized water, dried in a dehumidifier chamber overnight, and coated with 5 nm of osmium prior to imaging.

### Absorbance measurements

Absorbance spectra of AuNP–IgG conjugate solution with and without GoldEnhance^TM^ Blots solution were obtained using a NanoDrop UV–Vis spectrophotometer (Thermo Scientific, MA). AuNP–IgG conjugate solution was prepared as described in the “*Preparation of AuNP-conjugated IgG antibody”* above. Gold enhancement treatment was carried out by centrifuging 500 μL of AuNP–IgG conjugate solution at 5000 × *g* for 10 min, removing 250 µL of supernatant, and resuspending the precipitate in 250 μL of GoldEnhance^TM^ Blots solution.

### Optical transmittance measurements

Transmittance spectra of nitrocellulose paper coated with AuNP–IgG conjugates treated with and without gold enhancement solution were obtained using a Perkin Elmer Lambda-900 UV–Vis–NIR spectrometer with an integrating sphere. Transmittance spectra of unmodified nitrocellulose paper were also obtained and used as a blank control. Nitrocellulose paper was cut into 20 × 20 mm pieces using a CO_2_ laser cutter and immersed in AuNP–IgG conjugate solution with or without GoldEnhance^TM^ Blots solution for 5 s and dried in a dehumidifier chamber for 1 h prior to measurements.

### Proof-of-concept demonstration

One-week-old pig cadavers stored at −20 °C were obtained from a local swine farm. A 30 × 30 mm section of the cadaver was rinsed with deionized water and shaved using a razor. One hundred microliters of interstitial fluid simulant spiked with 1024 ng/mL *Pf*HRP2 or PBS was dermally injected into a 1-cm^2^ area of the skin section using a syringe pump (BD Scientific, NJ) to mimic the interstitial fluid content in unfrozen porcine cadaver skin (~150 µL/cm^2^)^41^. The patch was applied to the skin and pressed gently to initiate interstitial fluid extraction. After ~15 min, 30 μL of PBS was applied to the buffer port. Photographs of the test results were obtained using an iPhone XS.

## Supplementary information

Supplementary Information

## Data Availability

All data needed to evaluate the conclusions in the paper are present in the paper and/or the Supplementary Materials. Additional data related to this paper may be requested from the authors.

## References

[1] Kassebaum, N. J. et al. Global, regional, and national disability-adjusted life-years (DALYs) for 315 diseases and injuries and healthy life expectancy (HALE), 1990–2015: a systematic analysis for the Global Burden of Disease Study 2015. *Lancet* **388**, 1603–1658 (2016). 10.1016/S0140-6736(16)31460-X PMC5388857 27733283

[2] World Health Organization. *Global Health TB Report* (WHO, 2018).

[3] World Health Organization. *World Health Statistics 2018: Monitoring Health for the SDGs, Sustainable Development Goals* (WHO, 2018).

[4] World Health Organization. *World Malaria Report 2018* (WHO, 2018).

[5] Mabey D, Peeling RW, Ustianowski A, Perkins MD (2004). Diagnostics for the developing world. Nat. Rev. Microbiol..

[6] Geaghan SM (2014). Infection transmission associated with point of care testing and the laboratory’s role in risk reduction. J. Int. Fed. Clin. Chem. Lab. Med..

[7] Wilson NO, Adjei AA, Anderson W, Baidoo S, Stiles JK (2008). Detection of *Plasmodium falciparum* histidine-rich protein II in saliva of malaria patients. *Am*. J. Trop. Med. Hyg..

[8] Oguonu T (2014). The performance evaluation of a urine malaria test (UMT) kit for the diagnosis of malaria in individuals with fever in south-east Nigeria: cross-sectional analytical study. Malar. J..

[9] Oyibo WA (2017). Multicenter pivotal clinical trial of urine malaria test for rapid diagnosis of *Plasmodium falciparum* malaria. J. Clin. Microbiol..

[10] Fung AO (2012). Quantitative detection of PfHRP2 in saliva of malaria patients in the Philippines. Malar. J..

[11] Kim Y-C, Park J-H, Prausnitz MR (2012). Microneedles for drug and vaccine delivery. Adv. Drug Deliv. Rev..

[12] Gill HS, Denson DD, Burris BA, Prausnitz MR (2008). Effect of microneedle design on pain in human subjects. Clin. J. Pain..

[13] Ventrelli L, Marsilio Strambini L, Barillaro G (2015). Microneedles for transdermal biosensing: current picture and future direction. Adv. Healthc. Mater..

[14] Chang H (2017). A swellable microneedle patch to rapidly extract skin interstitial fluid for timely metabolic analysis. Adv. Mater..

[15] He R (2020). A hydrogel microneedle patch for point-of-care testing based on skin interstitial fluid. Adv. Healthc. Mater..

[16] Wang PM, Cornwell M, Prausnitz MR (2005). Minimally invasive extraction of dermal interstitial fluid for glucose monitoring using microneedles. Diabetes Technol. Ther..

[17] Miller, P. R. et al. Extraction and biomolecular analysis of dermal interstitial fluid collected with hollow microneedles. *Commun. Biol*. **1**, 1–11 (2018). 10.1038/s42003-018-0170-z PMC6197253 30374463

[18] Windmiller JR (2011). Microneedle array-based carbon paste amperometric sensors and biosensors. Analyst.

[19] Miller PR (2012). Multiplexed microneedle-based biosensor array for characterization of metabolic acidosis. Talanta.

[20] Chua B, Desai SP, Tierney MJ, Tamada JA, Jina AN (2013). Effect of microneedles shape on skin penetration and minimally invasive continuous glucose monitoring in vivo. Sens. Actuators, A Phys..

[21] Invernale MA (2014). Microneedle electrodes toward an amperometric glucose-sensing smart patch. Adv. Healthc. Mater..

[22] Nemani KV, Moodie KL, Brennick JB, Su A, Gimi B (2013). In vitro and in vivo evaluation of SU-8 biocompatibility. Mater. Sci. Eng. C..

[23] Otberg N (2004). Variations of hair follicle size and distribution in different body sites. J. Invest. Dermatol..

[24] Tan CY, Statham B, Marks R, Payne PA (1982). Skin thickness measurement by pulsed ultrasound; its reproducibility, validation and variability. Br. J. Dermatol..

[25] Wang PC, Paik SJ, Kim SH, Allen MG (2014). Hypodermic-needle-like hollow polymer microneedle array: fabrication and characterization. J. Microelectromechanical Syst..

[26] Davis SP, Landis BJ, Adams ZH, Allen MG, Prausnitz MR (2004). Insertion of microneedles into skin: measurement and prediction of insertion force and needle fracture force. J. Biomech..

[27] Jacobi U (2007). Porcine ear skin: an in vitro model for human skin. Ski. Res. Technol..

[28] Walther F (2007). Stability of the hydrophilic behavior of oxygen plasma activated SU-8. J. Micromech. Microeng..

[29] Delplanque A (2014). UV/ozone surface treatment increases hydrophilicity and enhances functionality of SU-8 photoresist polymer. Appl. Surf. Sci..

[30] Marquart L, Butterworth A, McCarthy JS, Gatton ML (2012). Modelling the dynamics of *Plasmodium falciparum* histidine-rich protein 2 in human malaria to better understand malaria rapid diagnostic test performance. Malar. J..

[31] Njoki PN (2007). Size correlation of optical and spectroscopic properties for gold nanoparticles. J. Phys. Chem. C..

[32] Jain PK, Lee KS, El-Sayed IH, El-Sayed MA (2006). Calculated absorption and scattering properties of gold nanoparticles of different size, shape, and composition: applications in biological imaging and biomedicine. J. Phys. Chem. B.

[33] Tran BQ (2018). Proteomic characterization of dermal interstitial fluid extracted using a novel microneedle-assisted technique. J. Proteome Res..

[34] Müller AC (2012). A comparative proteomic study of human skin suction blister fluid from healthy individuals using immunodepletion and iTRAQ labeling. J. Proteome Res..

[35] Lee KT (2014). Capture of the circulating *Plasmodium falciparum* biomarker HRP2 in a multiplexed format, via a wearable skin patch. Anal. Chem..

[36] Hendriksen ICE (2013). Defining falciparum-malaria-attributable severe febrile illness in moderate-to-high transmission settings on the basis of plasma PfHRP2 concentration. J. Infect. Dis..

[37] Kool J (2007). Suction blister fluid as potential body fluid for biomarker proteins. Proteomics.

[38] Ribet, F. et al. Minimally invasive and volume-metered extraction of interstitial fluid: bloodless point-of-care sampling for bioanalyte detection. *DiVA***diva2**, 1388020 (2020) http://www.diva-portal.org/smash/record.jsf?pid=diva2%3A1388020;delimiter=8890.

[39] Pleitez M, Von Lilienfeld-Toal H, Mäntele W (2012). Infrared spectroscopic analysis of human interstitial fluid in vitro and in vivo using FT-IR spectroscopy and pulsed quantum cascade lasers (QCL): establishing a new approach to non invasive glucose measurement. Spectrochim. Acta - Part A Mol. Biomol. Spectrosc..

[40] Chaudhary A, McShane MJ, Srivastava R (2010). Glucose response of dissolved-core alginate microspheres: towards a continuous glucose biosensor. Analyst.

[41] Samant PP, Prausnitz MR (2018). Mechanisms of sampling interstitial fluid from skin using a microneedle patch. Proc. Natl Acad. Sci. USA.

