# Supplementary material for: Microneedle-based skin patch for blood-free rapid diagnostic testing
Source: Microsyst Nanoeng. 2020 Nov 2;6:96. doi: 10.1038/s41378-020-00206-1 (PMC7605440; doi:10.1038/s41378-020-00206-1)
Supplement: Supplementary file 1 — Supplementary Information [file 41378_2020_206_MOESM1_ESM.docx]

**Supplementary Information**

**Microneedle-based skin patch for blood-free rapid diagnostic testing**

Xue Jiang^1^ and Peter B. Lillehoj^1, 2^*

^1^Department of Mechanical Engineering, Rice University, Houston, TX 77005, USA.

^2^Department of Bioengineering, Rice University, Houston, TX 77030, USA.

*Corresponding Author: Peter B. Lillehoj; Tel: 713-348-7344; E-mail: lillehoj@rice.edu


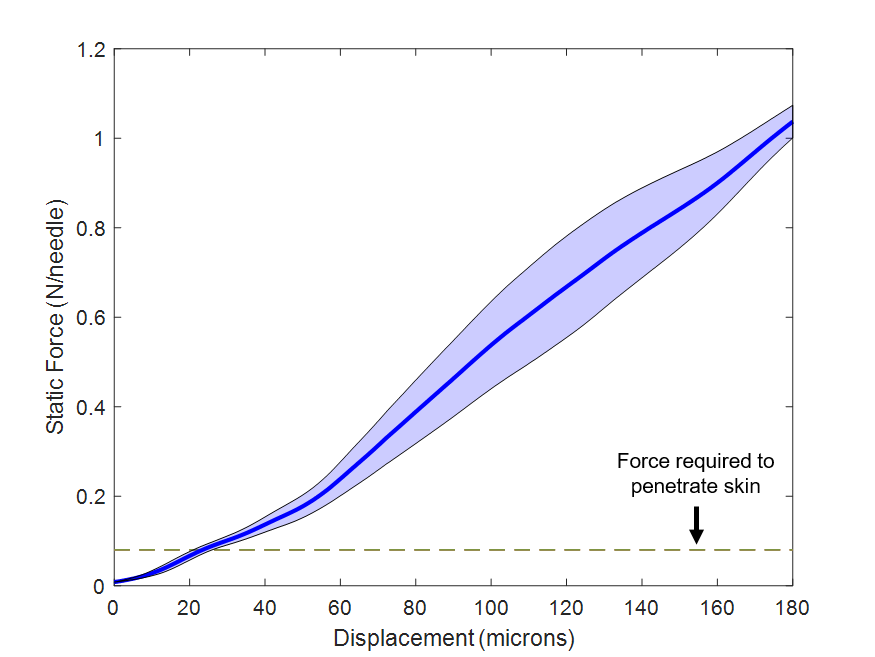


**Figure S1: Microneedle mechanical testing**. Compression force *vs*. displacement curves for the 4 × 4 microneedle array. The solid line represents the mean values ± standard deviation (shaded region) obtained from four different microneedle arrays.


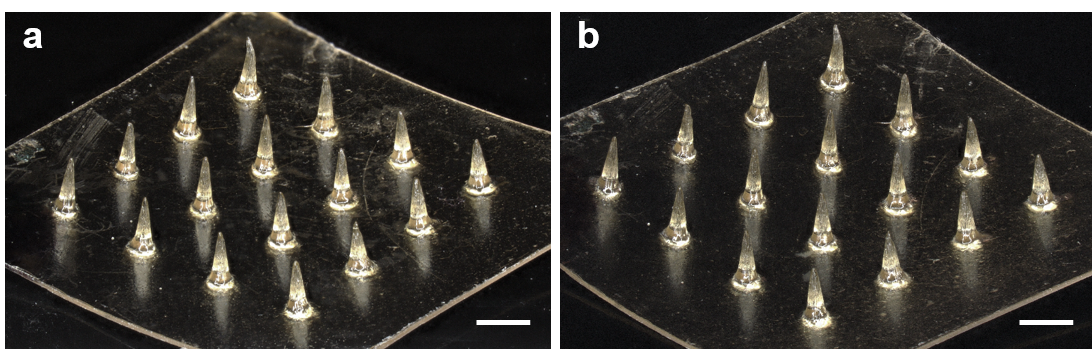


**Figure S2: Microneedle integrity following skin insertion.** Optical micrographs of the microneedle array before (**a**) and after (**b**) insertion in porcine skin. Scale bar, 1 mm.


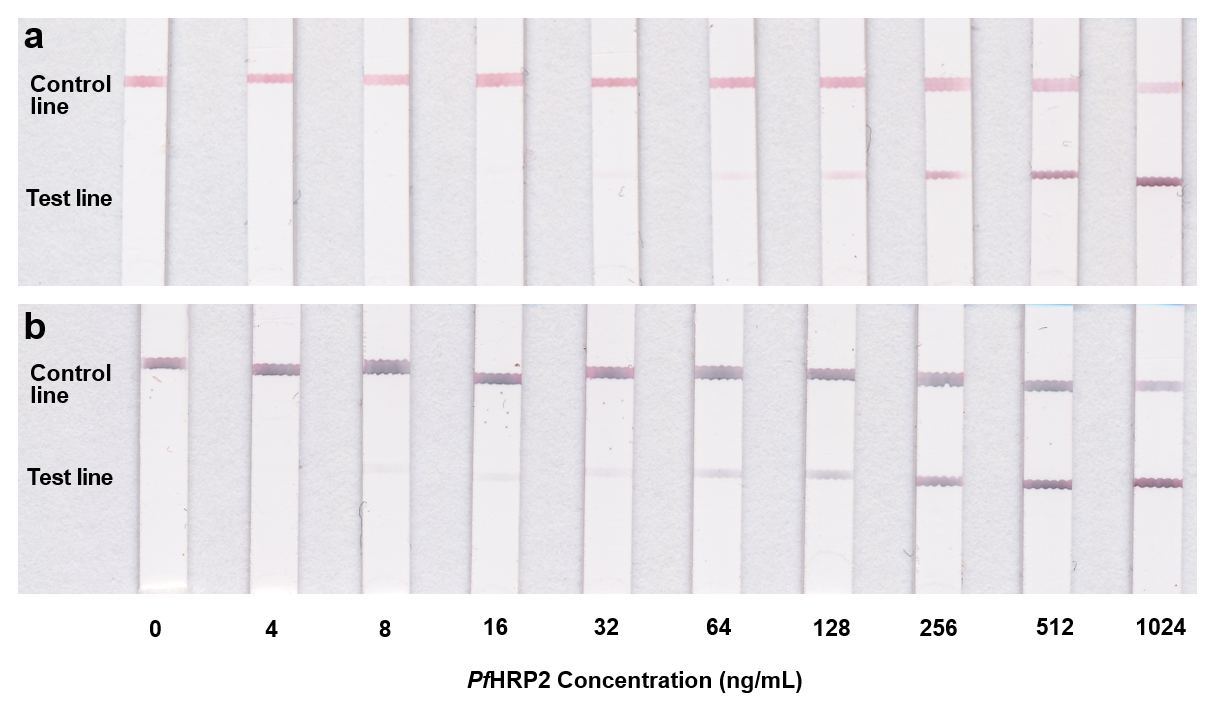


**Figure S3: Sensitivity of the *Pf*HRP2 assay with and without gold enhancement.** (**a**) Test results of interstitial fluid containing increasing concentrations of *Pf*HRP2 without gold enhancement treatment. (**b**) Test results of interstitial fluid containing increasing concentrations of *Pf*HRP2 with gold enhancement treatment.


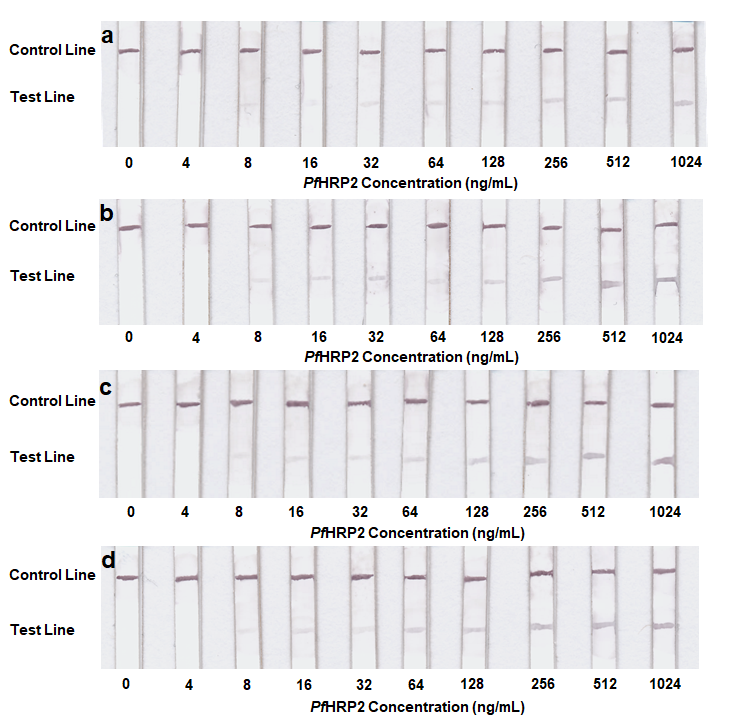


**Figure S4: Sensitivity of the *Pf*HRP2 assay with varying sample volumes.** Test results of (**a**) 2 μL, (**b**) 5 μL, (**c**) 10 μL and (**d**) 15 μL of interstitial fluid containing increasing concentrations of *Pf*HRP2 with gold enhancement treatment.


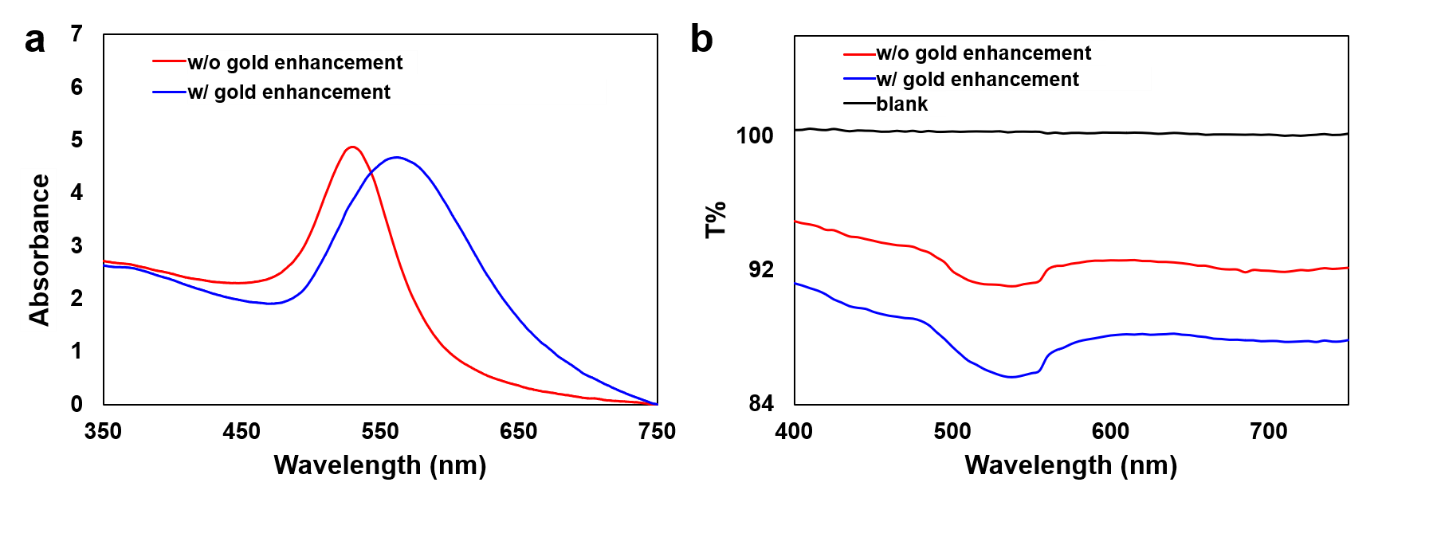


**Figure S5: Optical characterization of AuNP-IgG conjugates with and without gold enhancement.** (**a**) Absorbance spectra of AuNP-IgG conjugates with and without gold enhancement treatment. (**b**) Optical transmittance spectra of unmodified nitrocellulose paper (blank) and nitrocellulose paper containing AuNP-IgG conjugates with and without gold enhancement treatment.


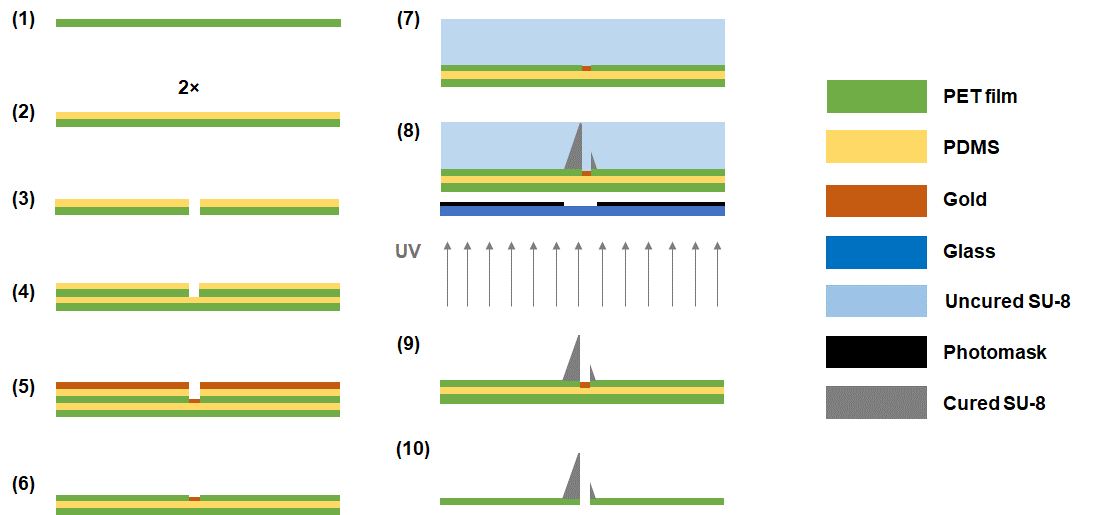


**Figure S6: Microneedle array fabrication process.** Schematic illustration of the fabrication process for generating hollow SU-8 microneedle arrays.
